# Reconstructing social networks of Late Glacial and Holocene hunter–gatherers to understand cultural evolution

**DOI:** 10.1098/rstb.2020.0318

**Published:** 2022-01-31

**Authors:** Valéria Romano, Sergi Lozano, Javier Fernández-López de Pablo

**Affiliations:** ^1^ Instituto Universitario de Investigación en Arqueología y Patrimonio Histórico (INAPH), Universidad de Alicante, Edificio Institutos Universitarios, 03690 San Vicente del Raspeig, Alicante, Spain; ^2^ Departament d'Història Econòmica, Institucions, Política i Economia Mundial, Universitat de Barcelona, Avinguda Diagonal 690, 08034 Barcelona, Spain; ^3^ Universitat de Barcelona Institute of Complex Systems (UBICS), Universitat de Barcelona, Martí Franqués 1, 08028 Barcelona, Spain

**Keywords:** human cultural evolution, socio-spatial networks, cultural transmission, evolutionary archaeology

## Abstract

Culture is increasingly being framed as a driver of human phenotypes and behaviour. Yet very little is known about variations in the patterns of past social interactions between humans in cultural evolution. The archaeological record, combined with modern evolutionary and analytical approaches, provides a unique opportunity to investigate broad-scale patterns of cultural change. Prompted by evidence that a population's social connectivity influences cultural variability, in this article, we revisit traditional approaches used to infer cultural evolutionary processes from the archaeological data. We then propose that frameworks considering multi-scalar interactions (from individuals to populations) over time and space have the potential to advance knowledge in cultural evolutionary theory. We describe how social network analysis can be applied to analyse diachronic structural changes and test cultural transmission hypotheses using the archaeological record (here specifically from the Marine Isotope Stage 3 *ca* 57–29 ka onwards). We argue that the reconstruction of prehistoric networks offers a timely opportunity to test the interplay between social connectivity and culture and ultimately helps to disentangle evolutionary mechanisms in the archaeological record.

This article is part of a discussion meeting issue ‘The emergence of collective knowledge and cumulative culture in animals, humans and machines’.

## 1. Introduction

Culture is believed to have played an important role in the social evolution of humans, and technology, for its part, has been key to their evolutionary success. The backbone of this argument is that the selection of inheritable social phenotypes would not have been possible without variations in individuals' social strategies. It is the transmission of social/cultural characters, in which individuals copy or learn from others, that produces evolutionary changes [1]. Because the use of social information enables individuals to adjust their behaviour faster to changing ecological circumstances [2,3], social transmission is a major mechanism of cultural evolution. Therefore, two major questions are of interest to cultural evolutionists: (i) how does individual social behaviour affect cultural transmission; and (ii), what are the consequences of these microscale mechanisms for the long-term patterns of cultural accumulation or loss?

Multiple evidence has shown that individual social decisions (i.e. with whom and how frequently to interact) affect population-level outcomes, such as cultural transmission [4–6]. This interplay between social connectivity and culture highlights the need to consider multi-scalar processes to investigate cultural evolution. On the one hand, we can address individual behaviour by conducting field (e.g. [7]) and laboratory (e.g. [8]) experiments, and on the other, we can take into account population patterns based on direct empirical observations and/or simulations (e.g. [9,10]). The current challenge is to explain cultural variation at the population-level while maintaining a firm grasp on evolutionary processes at different spatial and temporal scales. From this perspective, archaeology holds a huge potential to decipher long-term evolutionary patterns of cultural changes [11]. The archaeological record enables us to track behavioural variation, and changes in social connectivity, over thousands of years under different socio-environmental pressures (e.g. climatic and environmental changes, demographic variations). Yet, few studies have investigated the influence of social connectivity on cultural transmission in human prehistory [12]. Reasons may include the intrinsic limitations of the archaeological data (e.g. [13]); the difficulty (and often the impossibility) of obtaining individual-level data and the need for concepts and tools to address the interplay between social connectivity and long-term cultural dynamics.

In this manuscript, we revisit the main approaches used to infer evolutionary processes from archaeological data. This allows us to propose state-of-the-art methodologies to estimate past human interaction patterns. Specifically, we argue that we can gain important insights into cultural transmission mechanisms by implementing modelling approaches to formally analyse prehistoric hunter–gatherer socio-spatial networks (i.e. a mathematical quantification and graphical representation of social connectivity and spatial distribution patterns). The network analysis methodology considers distinct levels of interactions (from individual to population-level). The latter enables integrating empirical approaches and structural comparisons, thus providing a deeper understanding of cultural transmission processes. Because our methodological approach relies on the reconstruction of socio-spatial networks, its application is restricted to periods of human prehistory in which the empirical evidence (e.g. number of archaeological sites, material culture) holds sufficient resolution to trace diachronic changes in both cultural behaviour and social connectivity. While the examples discussed throughout this manuscript come from the Late Glacial and Holocene hunter–gatherers (Marine Isotope Stage (MIS) 1, *ca* 11.7 ka onwards), its application may also be feasible to older periods (MIS 2, *ca* 29–14 ka and MIS 3 57–29 ka). We believe this approach provides new avenues to study cultural evolution in human prehistory.

## 2. Structural organization of prehistoric hunter–gatherers

Prehistoric hunter–gatherers were organized into a fluid multi-level social structure [14]. This means that bands, the basic unit of social organization, were nested, forming regional groups (or maximum bands). This configuration characterizes a multi-level social structure [15] in which individuals from different bands, but from the same regional group, socially interacted and shared information to a greater degree than individuals from different regional groups. In mobile foragers, bands were usually composed of a variable number of individuals (between 20 and 40), from different family units [15,16]. They regularly moved between groups of different size and density—which implied variations on organizational arrangements [17]. For example, Wengrow & Graeber [18] argued that Upper Palaeolithic societies had flexible political organizations, which encompassed both hierarchical (i.e. positions by authority) and egalitarian modes.

Based on an extensive review of ethnographic data, we can assume that Upper Palaeolithic and Mesolithic social organizations would be comparable with the documented cases of extant hunter–gatherers [19]. The use of well-documented ethnographic data in different environmental contexts allow us to test hypotheses about the relationship between demographic and environmental variables (e.g. climate instability, fluctuation in carrying capacity) using the archaeological record [19]. For example, Binford [19] proposed a density packing threshold that would not surpass 3.4 or 17.9 persons 100 km^−2^ if the mobile people depended upon terrestrial animals or if they exploited aquatic resources, respectively. Another example comes from a study comparing the structure of 1189 social groups in 339 extant hunter–gatherers: they showed an organization in hierarchical, self-similar networks of predictable group size [20]. Researchers can then approximate the spatial dimensions based on two considerations: the *minimum-band* size refers to the number of people that would guarantee the presence of mates (i.e. a population breeding unit). *Maximum-band* size refers to the total number of ‘people which can be consistently integrated by the cultural mechanisms of a given cultural system and which is consistently required for the successful operation of such a cultural system’ [16, p. 1974]. Based on simulations about the use of space and potential resource exploration, Wobst [16] proposed that Palaeolithic maximal bands would have ranged between 175 and 300 individuals under favourable conditions. Minimal bands were not viable social institutions in the long term, so they have been considered as a series of adjacent band territories—whose territorial size and range varied according to local resources [21].

The dynamics of hunter–gatherer populations, as any other biological population, depended on the influence of climatic effects on resource availability—which ultimately influenced the carrying capacity of the environment. At higher latitudes, to sustain large group sizes but low population densities, human groups developed patterns of fission and fusion to adjust their foraging patterns [22]. The archaeological record shows a broad array of evidence demonstrating the link between shifts in climate and environmental productivity with changes in population size and densities [23], both at continental [24,25] and regional scales [26–28]. In Atlantic Iberia, a recent example concerning hunter–gatherers of the Late Glacial and Early Holocene showed an increase in population size and density during the Late Mesolithic period. This was concomitant to a higher reliance on aquatic resources, prompting the population to cluster on ecologically rich estuaries [29]. Presumably, changes in foraging strategies and sociality may have provided an optimal condition for the emergence of human cumulative culture [30]. These examples reinforce the assumption that significant changes in settlement patterns and distribution translate to changes in patterns of social interactions, and, therefore, on socio-spatial networks. Put simply, environmental pressures (like drastic climatic shifts) influence demographic patterns (such as population size), which in turn may cause variation in socio-spatial connectivity [12]. We then expect that social networks may be sensitive to demographic changes caused by migration activity, shifts in population growth, among others.

One hypothesis is that social networks were formed and maintained owing to the need to exchange information, which in turn allowed mitigating risks and resource uncertainties, creating ‘safety nets’ [21]. Whallon [21] introduced the concept of non-’utilitarian’ mobility, stating that long-distance exchanges of non-utilitarian items, such as personal ornaments, were indicators of social interactions on a much broader social scale (beyond bands). He concluded that changes in long-distance movements from the Upper Palaeolithic to the Mesolithic in southwestern Germany could be explained, partially at least, by temporal and spatial variations in resource availability, which affected the organization and mobility of prehistoric hunter–gatherers [21]. In this context, Fitzhugh *et al.* [31] developed an information network model that predicted the degree and intensity of information exchange as a result of environmental unpredictability and connection costs—following the theoretical framework proposed by Whallon [21]. Among their conclusions, they argued that hunter–gatherer bands would have been more connected at ‘local, intergroup, regional and supraregional scales when the costs of networking are low and environmental productivity and predictability are also low’ [31, p. 85]. Altogether, the organization of information networks at different scales (i.e. inter-band, supra-band and regional network) varied across gradients of spatial connectivity, environmental productivity and resource base predictability [31]. It is, therefore, imperative to keep in mind that Late-Middle/Upper Palaeolithic and Mesolithic societies were not compartmentalized: they were in fact connected through movement and information exchange.

## 3. The scope for assessing cultural evolution in prehistoric archaeology

The use of evolutionary approaches to investigate cultural changes is relatively recent in archaeology [32–34]. The seminal works of Cavalli-Sforza & Feldman [35] and Boyd & Richerson [36] introduced the application of techniques belonging to behavioural ecology, genetics and population biology to understand cultural transmission. These studies highlight how information, like genes, is transmitted from parents to offspring (i.e. vertical transmission). Yet, unlike genetic inheritance, information can also be transmitted among unrelated peers (i.e. horizontal transmission) and/or from one generation to another younger generation (i.e. oblique transmission) [36]. The parallels between genetic and cultural inheritance have been well-established and are explained in detail elsewhere [37]. In this section, we focus on how researchers infer evolutionary processes from the archaeological record.

First, it is important to recall some of the components of cultural evolutionary theory. In a nutshell, cultural evolution rests on the creation of new traits (i.e. innovation), on their transmission through social contacts (e.g. through imitation and active teaching) and on forces that may include (e.g. learning mistakes) or reduce (i.e. biased transmission) trait variability. As previously explained, forms of cultural transmission includes horizontal, vertical and oblique transmission while well-known models of transmission include conformity (i.e. individuals adopt the most common cultural trait) and others with transmission biases [36]. These processes are complex and challenging to infer from archaeological data, given their coarse chronological resolution and incomplete nature. Therefore, the question is: to what extent is it possible to infer microscale phenomena when the resulting archaeological record is an aggregation of events over time, such as snapshots of event superpositions? Shennan [38] called it the ‘inverse problem’: ‘as archaeologists have to infer microscale processes producing a pattern from the pattern itself’ [38, p. 1071]. It was in this context that O'Brien & Lyman [39] proposed a procedure for evolutionary archaeological approaches. They state the need to (i) demonstrate a relationship between ancestor and descendant to build a history of transmission (e.g. using artefact chronology and similarity indexes), and (ii) to identify the forces causing variation in the archaeological record (e.g. using the frequency of discrete traits to test models of random copying [40]; see [38] for a comprehensive discussion on the topic).

### (a) Approaches to identify cultural changes in the archaeological record

In anthropology, and by extension in archaeology, the terms cultural and/or technological trait, manifested in artefacts/material culture, are used to refer ‘to a unit of transmissible information that encodes behavioural characteristics of individuals or groups' [41, p. 693]. By tracking variations of those traits in the material culture found in the archaeological record (e.g. lithic raw materials, ceramic composition and projectile-point morphology) through time and space, it is possible to identify changes in cultural patterns. In particular, the most ubiquitous artefact category left by prehistoric hunter–gatherers is lithic tools, which in most cases are related to food-getting activities. Because lithic materials are omnipresent and well-preserved across time, patterns of variation in artefact shapes and tool-making techniques can be inferred in great detail. Importantly, the production of lithic artefacts is a type of knowledge that is acquired through social learning. In this way, it favours the detection of the inter-generational variability of socially transmitted technologies. Therefore, the archaeological record, and specifically, hunter–gatherer subsistence tools, hold enormous potential to understand human behavioural diversity and cultural evolution.

The two main approaches used to infer evolutionary processes from the archaeological data are cladistics (i.e. the identification of the possible phylogenetic relationship between artefacts) and artefact variability (i.e. the quantification of artefact variation to identify learning biases) [11]. Cladistics is used to identify the hypothetical relationship between classes of prehistoric technology considering the most recent common ancestry. This approach considers that artefacts will share specific characters (i.e. ‘homologous artefacts’) and that new structures and functions arise from the modification of existing traits. For example, O'Brien *et al.* [42] used Clovis point manufacture to reconstruct relationships among eastern North American Palaeoindian points. At the time, it was unclear whether the continental-wide occurrence of fluted-point forms represented a single cultural expression. Results indicated that much of the variation in the form (e.g. tang-tip shape and outer tang angle) was related to the hafting elements of a point, but other characters did not change or did so moderately (e.g. base shape). Overall results suggested that there was both temporal and spatial patterning of some of the projectile-point classes [42]. These results highlighted the application of cladistic methods to unravel the history of transmission in the archaeological record. As in the biological sciences, the parsimony argument is that if a cultural trait of similar form is found in a geographically connected area, we may assume inheritance from a common ancestor. If these traits are found in disconnected places, convergent evolution may be a plausible explanation [43,44]. The cladistic approach, however, presents two limitations: (i) it precludes the emergence of similar artefacts owing to similar selective pressures; and (ii), it only considers the possibility of vertical transmission [41]. These pragmatic assumptions resulted in a heated debate, but cladistics remains an important methodological and theoretical basis to identify evolutionary relationships in the archaeological record [33].

The quantification of artefact variability, centred here on technological and morphometrics variation, is also used to study cultural evolution in archaeology [11]. Originally, archaeologists quantified artefact variability over time and space in order to interpret human behaviour—this being central in culture-historical reconstructions. With the advent of cultural evolutionary theory, however, it is now possible to test hypotheses about this variation. Technological and morphometric variation of artefacts can be traced in the archaeological record through inter-generational time scales. Because, in small-scale societies, the transmission of tool making and tool use knowledge usually happens in contexts of social learning, the analysis of artefact variation through time can be used to test specific models of cultural transmission (conformist, biased, etc.). Within this field, the main assumption is that different cultural transmission processes leave distinct signatures in terms of artefact variations. Likewise, similar artefact forms would represent a link in the transmission chain. For example, some evolutionary archaeologists adapted neutral drift models to account for variation in the archaeological record. Eerkens & Bettinger [45] used Great Basin flaked-stone projectile points to examine the relationship between artefact variation and different transmission modes. Of the 52 type-attributes considered, only 11 exhibit variation. This low artefact variability in large geographical areas indicates that little error occurred in the process of cultural transmission [45]. The example illustrated how artefact variation can be used to test models of cultural transmission.

Another methodological approach includes morphometric analysis, such as two- and three-dimensional imaging. These methods use digital models to understand variation in material culture size and shape across time and/or space—especially in the context of evolutionary archaeology. This exciting field has generated consistent knowledge about the function, development and evolution of material culture, but its application has been mostly restricted to lithic artefacts [46]. One example includes the estimation of social interactions in the Pleistocene North America, based on bifacial asymmetry of projectile-point styles. Researchers discovered a temporal variation in flake scar patterning, suggesting the start of regionalization among New World colonists [47]. In summary, the main methodological approaches currently applied by evolutionary archaeologists are based on the identification of material culture similarities (e.g. manufacture techniques and morphometry). This provides a proxy to study evolutionary process based on the transmission of characters from one generation to another.

While novel methodological approaches allow an explicit investigation of cultural transmission processes in the archaeological record, its application often confronts cultural-history systematics based on traditional archaeological practice. For instance, considering the chronological period of interest in this manuscript (Late-Middle/Upper Palaeolithic and Mesolithic), the construction of cultural taxonomies (i.e. ‘the definition and description of taxonomic units that group assemblages according to their material culture and geographical and chronological distributions' [48, p. 1350]) has been strongly conditioned by national and regional historiographic traditions. This practice introduces a compartmentalization of the material culture (into discrete sub-systems) instead of considering the dynamic processes that led to the spatial–temporal distribution of artefact variability in the archaeological record. It also implies a series of limitations, including a methodological heterogeneity underlying the construction and interpretation of cultural taxonomies [48]. A novel research perspective focused on a population-based rethinking of artefact variability is starting to overcome these shortcomings, and it can significantly contribute to creating more robust archaeological systematics to be reconciled with archaeogenetic and palaeoenvironmental datasets [49,50].

This highlights the importance of several state-of-the-art methodologies to integrate evolutionary approaches to archaeology. Given the complex social organization of prehistoric hunter–gatherers and the dynamics of cultural transmission, an approach that considers social structural comparison and multi-scalar interactions (from individuals to populations) over time and space has the potential to advance knowledge in cultural evolutionary theory. In the following sections, we explore how well-developed methods for social structure analysis can increase our understanding of the selective forces shaping human cultural evolution.

## 4. Reconstructing prehistoric social networks to investigate cultural transmission

New analytical approaches to test the diachronic process of cultural transmission include social network analysis (SNA). SNA is a sophisticated methodology that allows a fine-scale assessment of patterns of interactions. It thus presents a great potential to unravel ecological and evolutionary processes (such as those found in animal biology: cooperation [51] and convention [52]). Although prominent in multiple fields (e.g. behavioural ecology, evolutionary psychology), SNA has only recently been applied in archaeology [53–55]. In a network of contacts, nodes usually correspond to archaeological sites, as a proxy for residential units (camps, villages or cities) and linkages between these nodes can be estimated through cultural similarity and travelling distance, among others. Owing to the spatial and chronological scales in which we can interpret social interactions using the prehistoric archaeological proxies, individual-level data is rare and scientists mostly focus on macro-regional interactions. Therefore, a major goal of reconstructing prehistoric social networks is to infer chronological changes through time and space in order to investigate the relationships between social networks and cultural changes.

Within this context, we, recently, proposed an integrative framework that outlines the dynamic relationship between environmental pressures, palaeodemography, socio-spatial structure and cultural transmission in human societies [12]. We claimed that the interplay between social structure and culture still needed to be incorporated in archaeology, and we detailed how SNA could be applied to investigate long-term patterns of cultural transmission in prehistoric archaeology. It is important to highlight here that macro-cultural phenomena have different temporal scales. As a result, the nature of social interactions is relevant (e.g. the distinction between intra/inter-band social interaction or migration) to evaluate the multi-scalar nature of human–environmental interactions and cultural changes.

To identify the impact of social connectivity on cultural transmission, several metrics exist that can be used to characterize a network's structural properties. One can classify them into node and global metrics, and each can be used to test a distinct facet of cultural transmission. These metrics are explained in detail elsewhere [56,57], but we will briefly introduce here some examples in the context of cultural evolution. First, we will focus on node metrics. The most well-known metrics are related to node centrality, which characterizes a node's direct and indirect connections. For example, *strength* determines the total intensity of links among nodes and *betweenness* refers to the relative importance of intermediate nodes in indirectly connecting others. While strength centrality can be used to assess a node's probability of obtaining and transmitting culture from its immediate contacts, betweenness centrality is important to estimate cultural transmission to peripheral nodes, which would be otherwise disconnected. For example, in subdivided networks, indirect measures of centrality are of great relevance to indicate potential transmission across sub-networks. On the other hand, global metrics refer to general patterns of social networks. One of the prominent metrics is *modularity*, defined as the extent to which a network is divided into clearly differentiable modules [58]. Studies have shown that the degree to which a network is subdivided is directly related to network efficiency [59], with intermediate values of modularity leading to high values of social transmission [60]. In summary, network metrics indicate different facets of the cultural transmission processes, but it is important to bear in mind the underlying biological question (see [12] for a translation of network metrics to the context of prehistoric hunter–gatherers).

An exemplar case study applying SNA to investigate cultural changes comes from the Late Glacial hunter–gatherers in northern Europe. Approximately 13 000 BP, the Laacher See volcano erupted, in what is today western Germany, devasting nearby areas and leading to many years of cold temperatures [61,62]. The local population, known as the Federmesser culture, appears to have migrated to southwest Germany and France. Meanwhile, in southern Scandinavia, the Bromme culture emerged, characterized by a much simpler cultural repertoire than the Ferdermesser [63]. A potential explanation for the reduced technological complexity in Bromme culture was the disruption of social networks, caused by the volcano eruption, that led to demographically mediated loss of cultural capital [64]. Riede [64] reconstructed socio-spatial networks using exotic materials, such as shell ornaments, amber and animal figurines from approximately known raw material sources. Results showed a low density and subdivided network and the absence of long-distance social connections with their neighbours in the south. The application of network analysis identified the decline in material culture complexity and a post-eruption regionalization in northern Europe. Because of the low level of social connectivity, these forager populations could be most impacted by climatic and environmental changes [64].

### (a) Dealing with the inherent complex nature of the archaeological record

The archaeological record holds extraordinary potential to unravel long-term patterns of cultural changes. However, it requires careful consideration in terms of data limitations. The archaeological record is, by its very nature, incomplete: many materials have been lost owing to environmental degradation and the remains are conclusively a small part of past human activities. This partial representation leads to a series of well-known analytical challenges when addressing traditional archaeological questions, and consequently when applying SNA [65]. Perhaps the most evident challenge is the fact that it is only possible to interpret the archaeological record if based on the temporal aggregation of material culture. Therefore, the reconstruction of prehistoric hunter–gatherer networks represents an aggregation of social connections, which leads to a higher emphasis on consistently reproduced relationships across the period under study [13].

The selection of appropriate archaeological proxies to trace both changes in culture and social interaction patterns throughout space and time requires thorough consideration. Archaeological site locations and chronology provide the basis for building spatial and temporal networks whose structural properties can be compared. The analysis of spatial networks can be significantly enriched when considering material culture proxies, whether utilitarian artefacts, ornaments or art evidence—allowing us to establish links based on cultural similarities. For the time framework that we focus on this manuscript (human prehistory from the MIS 3 onwards) and the kind of processes under study (the relationship between social connectivity and cultural transmission), lithic artefacts are probably the best proxies to trace that relationship over inter-generational time scales. Tool making (including booth design and manufacture) is a fundamental kind of knowledge transmitted by social learning, leaving behind a material record that is persistent to use-discard or taphonomic processes.

It is critical to evaluate the robustness and reliability of the archaeological networks before testing the influence of networks on cultural transmission. This includes assessing the impact of missing nodes and evaluating the stability of sparse networks, among others [65]. The foremost consideration is that it may be almost impossible to reconstruct complete prehistoric networks. Consequently, the first step is to assess whether structural properties of the sampled network do or do not approximate the same aspects of the original network. For example, Gjesfjeld [13] used bootstrap simulation to evaluate the stability of node centralities in the hunter–gatherer networks on Kuril islands. His results demonstrated that sparse archaeological networks were stable over distinct network sizes. Particularly, the strong stability of degree and eigenvector centrality throughout the sampling intervals indicated that the Okhotsk culture network could be interpreted as representative of the original network [13].

Finally, a growing body of literature is demonstrating the feasibility of applying network analysis to the prehistoric context (e.g. [64,66]). This may be the result of considerable efforts to introduce SNA into archaeology [12,53–55,67,68] but also to the advance of modelling tools that allow better archaeological data resolution (e.g. chronological modelling [69]), the increased interest in hypothesis-driven approaches and training in quantitative archaeology. We, therefore, argue that prehistoric archaeologists now have the opportunity to deepen scientific knowledge of long-term cultural transmission processes. SNA provides novel insights to investigate the relationship between cultural transmission and social connectivity over time. Nevertheless, caution should be applied regarding data limitations and research feasibility.

## 5. Prehistoric social networks and macro-regional cultural phenomena

When studying cultural evolution, it is important to remember that the stone age archaeological record suggests that some technological innovations appeared as punctuations after long static periods, with abrupt cultural changes that varied across time and space. The process was multifaceted and depended, among others, on environmental fluctuations, social factors (such as demography and migratory events) and learning opportunities [70–73]. In the previous section, we addressed the interplay between social structure and culture and argued that SNA can potentially help to build a clearer understanding of the conditions in which cultural changes occur. One example is a protocol based on the use of regional population estimates, combined with network analysis and multi-agent computational modelling, to decipher the relationship between demographic dynamics and cultural transmission in the Iberian Peninsula [34]. This protocol incorporates both social and spatial components to assess longitudinal variations in settlement networks affecting the overall connectivity among hunter–gatherer populations. Such a design was part of an effort to reconstruct population patterns (e.g. [26,29]) and offered a consolidated framework to study prehistoric long-term cultural patterns. It is based on a three-step methodology: (i) reconstruction of socio-spatial networks before and after the given temporal window; (ii) structural comparison of the networks based on the two snapshots; and (iii) performance of simulations of cultural dynamics on and of networks [34].

This explicit network-based approach can be applied to any macrophenomenon in which patterns of cultural change are linked to demographic variation. Classical examples include the emergence of innovations in small populations [74] and the loss of technological capital [70]—both representing the extremes of demographically dependent cultural transmission processes. In the Iberian Peninsula, two well-identified cultural macrophenomena in the archaeological record are the loss of technological capital during the Epipalaeolithic and the rapid spread of trapeze-based industries during the Late Mesolithic [75]. On the one hand, the rich Magdalenian traditions (i.e. portable art, curated bone and antler technologies) gradually disappeared during the Pleistocene–Holocene transition. On the other, trapezoid microliths spread to the Iberian Peninsula within a few generations during the Late Mesolithic period, at the end of the Early Holocene. These are examples of cultural changes that took place during the Late Glacial and Early Holocene in Europe, but many other macrophenomena exist that could be evaluated through the network-based approach (e.g. the symbolic use of shells and pigments). In order to analyse diachronic changes in social interactions, it is important to adopt a specific regional approach, temporal window and archaeological proxy. This contributes to consolidating a rather unexplored effect of prehistoric social networks on cultural transmission and to test evolutionary processes using explicit macro-regional models.

## 6. Predictions of cultural transmission in prehistoric hunter–gatherers

We generally expect cultural transmission to be dependent on social structure, but the degree to which cultural specialization, loss and/or diversification are a consequence of prehistoric social networks is still poorly understood. Considering the macroscale of prehistoric archaeological proxies, modes of cultural transmission are mostly restricted to horizontal and oblique ones. Vertical transmission would happen within families, but such a resolution is rarely detectable in the archaeological record. In a simplified scheme of cultural transmission modes, we could assume that horizontal and oblique transmissions expose individuals to more social learning opportunities than vertical transmission does. This would have consequences for cultural changes in the short term (a few generations) and long term (across several generations). If culture is hypothetically dependent only on vertical transmission, cultural changes should be slow, because individuals have access to a limited cultural pool. The contrary would be expected of individuals who learn from non-related conspecifics [49]. Therefore, our hypotheses are built around information exchange within and across bands/regional groups.

We may expect to observe in the temporal snapshots of the prehistoric data: (i) larger social networks during periods of relatively high population density; (ii) stronger links within regional groups; and (iii), changes in density and length covered by links according to resource distribution and availability (i.e. ecological scenario [21]). These are straightforward predictions in archaeology that mostly depend on population size and social connectivity. In terms of cultural evolution, we *need to investigate* whether: (iv) cultural loss happens in small and disconnected networks; and (v) cultural diversity is higher in mildly connected networks—as structural property seems to maximize both information transmission [60] and cultural accumulation [8,76]. These two combined features (high cultural diversity and maximized information transmission) would make these networks more resilient in the face of environmental perturbations—as people could rely on an extensive network of diverse contacts and information sources during periods of low resource availability [21]. These predictions are based on opportunities of social learning (access to skilled individuals), but they ultimately depend on individual predisposition and socio-environmental conditions (which may trigger innovations [77]). Here, we can assess how cultural information was expected to spread. However, another important and perhaps key question in evolutionary archaeology is the extent to which prehistoric hunter–gatherer social networks conditioned cumulative culture. Given our limited knowledge of individual innovation and transmission rates in prehistory, we can only discuss population-level outcomes. This may be sufficient to test hypotheses on the development of non-kin relationships (which is estimated by inter-bands interactions), the emergence of large and structured networks and finally on whether the unique human social structure is linked to reliance on cumulative culture.

Therefore, in order to reconstruct prehistoric networks, it is necessary to consider the levels of social interactions (whether intra- and/or inter-regional) and the archaeological proxy. For example, it has long been considered that personal ornaments (such as shells and fossil molluscs) could be traced to recreate networks that were socially motivated [21], and that the intensification of long-distance connections (over 300 km) could represent formations of alliances and safety nets [78]. Safety nets are understood as networks of contacts that allow people to move from their own area of scarcity to places where resources are available. This network may provide support during challenging periods [21], and maybe a driver of social connectivity [13]. Determining the underlying mechanisms of cultural transmission in prehistory helps us to understand the process of cultural evolution itself. We have thus outlined some of the conceptual frameworks allowing us to test network-based hypotheses and we hope this will lead to further research on the topic.

## 7. Conclusion

Our understanding of human cultural evolution would certainly benefit from the reconstruction of prehistoric socio-spatial networks. An increasingly large number of studies analyse the archaeological record from an evolutionary perspective, yet the effect of social connectivity on cultural transmission is still largely unknown. We highlighted that the temporal and spatial scales of social interactions are relevant to evaluate the multi-scalar nature of human–environmental interactions and cultural changes. SNA could help to decipher human cultural responses to changing environments. An obvious concern is whether the archaeological record is robust enough to formally reconstruct and analyse prehistoric hunter–gatherer social networks. Among the reasons to believe that the application of network analysis is timely, is the access to higher-quality archaeological data and state-of-the-art methodologies. The latter enables us to assess the reliability of these networks. By applying SNA, researchers are able to uncover relevant patterns of social interactions, and consequently obtain a better picture of the selective mechanisms underlying human cultural evolution.

## Data accessibility

This article has no additional data.
